# Two new endophytic species of *Parafenestella* (*Pleosporales*, *Cucurbitariaceae*) from Xizang, China, revealed by morphology and multi-locus phylogeny

**DOI:** 10.3897/mycokeys.137.197730

**Published:** 2026-07-20

**Authors:** Kaiqi Pang, Yingying Wu, Yingmei Liang, Ning Jiang

**Affiliations:** 1 The Key Laboratory for Silviculture and Conservation of the Ministry of Education, Beijing Forestry University, Beijing 100083, China Key Laboratory of National Forestry and Grassland Administration on Forest Ecosystem Protection and Restoration, Ecology and Nature Conservation Institute, Chinese Academy of Forestry Beijing China; 2 Museum of Beijing Forestry University, Beijing Forestry University, Beijing 100083, China The Key Laboratory for Silviculture and Conservation of the Ministry of Education, Beijing Forestry University Beijing China; 3 Key Laboratory of National Forestry and Grassland Administration on Forest Ecosystem Protection and Restoration, Ecology and Nature Conservation Institute, Chinese Academy of Forestry, Beijing 100091, China Museum of Beijing Forestry University, Beijing Forestry University Beijing China

**Keywords:** *
Ascomycota
*, endophytes, molecular phylogeny, new taxa, taxonomy, Tibetan Plateau

## Abstract

During a mycological survey of high-altitude forests in Xizang (Tibet), China, four endophytic fungal strains were isolated from the asymptomatic tissues of *Cupressus gigantea* and *Rosa sericea*. Based on morphological characteristics and multi-locus phylogenetic analyses of a five-gene dataset (ITS, LSU, *tub2*, *tef1*, and *rpb2*), these isolates are introduced as *Parafenestella cupressicola***sp. nov**. and *P. xizangensis***sp. nov**. (*Cucurbitariaceae*, *Pleosporales*). Phylogenetically, *P. cupressicola* forms a sister lineage to *P. ulmi* and *P. ulmicola* but differs in conidiogenous cell and conidia dimensions. *Parafenestella xizangensis* shares similar conidial morphology with *P. salicis*, but differs in molecular sequences. These findings expand the known host range and geographic distribution of *Parafenestella* and suggest that additional endophytic diversity remains to be discovered in alpine forest ecosystems.

## Introduction

*Cucurbitariaceae* (*Pleosporales*, *Dothideomycetes*), established by Winter (1885), is a species-rich group of fungi with a broad host range and a global distribution, primarily occurring in temperate and Mediterranean regions (Jaklitsch et al. 2018; Su et al. 2022). Members of this family are traditionally characterized by their immersed or erumpent clustered ascomata, which are often accompanied by a subiculum, and the production of brown muriform ascospores within bitunicate asci (Doilom et al. 2013; Jaklitsch and Voglmayr 2020). Their asexual morphs are typically coelomycetous, appearing as *Phoma*- or *Pyrenochaeta*-like pycnidia that produce hyaline conidia (Valenzuela-Lopez et al. 2018; Jaklitsch and Voglmayr 2020).

Ecologically, *Cucurbitariaceae* species exhibit diverse lifestyles: while most are saprobes found on the wood and bark of trees and shrubs, others are recognized as plant pathogens, fungicolous parasites, or even opportunistic pathogens of humans and animals (Wanasinghe et al. 2017; Jaklitsch and Voglmayr 2020; Eisvand et al. 2024; Zhang et al. 2024). Recent investigations have also confirmed the capacity of certain members to exist as endophytes within healthy plant tissues (Jaklitsch et al. 2018; Hill et al. 2023).

Within the phylogenetic framework of *Cucurbitariaceae*, the fenestelloid clades have historically posed significant taxonomic challenges due to their complex structure and convergent morphology (Jaklitsch and Voglmayr 2020). Based on a comprehensive multi-gene reassessment, Jaklitsch et al. (2018) redefined the family and established the genus *Parafenestella*, with *P. pseudoplatani* as the generic type. Species of *Parafenestella* typically form small groups or clusters of fewer than ten ascomata and were initially recognized for their specialized fungicolous habit on members of the *Diaporthales*, particularly the genera *Cytospora* and *Diaporthe* (Jaklitsch and Voglmayr 2020).

Ongoing global fungal surveys continue to expand the known ecological and geographic boundaries of *Parafenestella* (Ilyukhin et al. 2022; Su et al. 2022; Eisvand et al. 2024). For instance, Eisvand et al. (2024) introduced *P. quercicola* from leaf spots on *Quercus brantii* in the Zagrosian forests of Iran. Concurrently, Su et al. (2022) described three novel saprobic species from woody litter in northeast China: *P. changchunensis* on *Populus*, alongside *P. ulmi* and *P. ulmicola* on *Ulmus pumila*. Additionally, Ilyukhin et al. (2022) described *P. ontariensis* as a saprobe on *Acer negundo* in North America. These discoveries highlight the remarkable ability of *Parafenestella* to adapt to diverse hosts across different continental climates.

Accurate species delimitation in *Parafenestella* remains difficult based on morphology alone, as diagnostic characters are highly variable and often overlap among cryptic species (Jaklitsch and Voglmayr 2020). Consequently, molecular phylogeny has become indispensable for achieving taxonomic clarity. Multi-locus analyses have revealed that while ribosomal DNA markers like ITS and LSU show low resolution at the species level, protein-coding genes provide far superior diagnostic power. Notably, Assemble Species by Automatic Partitioning (ASAP) and comparative single-locus evaluations have identified *tub2* as the most informative DNA barcode for delimiting *Parafenestella* species, followed closely by *rpb2* and *tef1* (Su et al. 2022).

Despite these taxonomic and methodological advancements, the diversity of the *Cucurbitariaceae* in extremely high-altitude environments remains poorly understood. Xizang, China, possesses unique alpine forest ecosystems and high levels of endemism. However, the presence and diversity of *Parafenestella* as endophytes in Xizang have not yet been explored. During a recent survey of fungal resources in the forests of Xizang, four fungal strains were isolated from asymptomatic plant tissues. The aim of this study was to provide a detailed morphological description and a robust phylogenetic analysis based on a concatenated dataset of ITS, LSU, *tub2*, *tef1*, and *rpb2*.

## Materials and methods

### Sample collection and fungal isolation

Healthy, asymptomatic cones of *Cupressus gigantea* and branch bark of *Rosa sericea* were collected during field expeditions in the high-altitude environments of Xizang, China; their important collection information was noted (Rathnayaka et al. 2025) and brought back to Chinese Academy of Forestry (**CAF**) in Beijing. To isolate endophytic fungi, the collected plant samples were washed thoroughly with tap water and subjected to a strict surface-sterilization protocol: immersion in 70% ethanol for 1 min, followed by immersion in 2% NaOCl for 2 min, and finally rinsed three times in sterile distilled water. The surface-sterilized tissues were dried on sterile filter paper, cut into small segments, and plated onto potato dextrose agar (PDA) supplemented with streptomycin sulfate (50 μg/mL) to suppress bacterial growth. The plates were incubated at 25 °C in darkness and monitored daily for fungal emergence. Hyphal tips migrating from the plant tissues were transferred to fresh PDA to obtain pure cultures. Specimens were deposited in the Herbarium of the CAF, Beijing, China, and living cultures were deposited in the China Forestry Culture Collection Center (**CFCC**), Beijing, China.

### Morphological observation

To evaluate macroscopic culture characteristics, isolates were grown on PDA at 25 °C in the dark. Colony diameters, colors, and textures were recorded over two weeks. To induce sporulation, isolates were inoculated onto Synthetischer Nährstoffarmer Agar (SNA) supplemented with sterile pine needles. The development of pycnidia on the SNA medium was monitored using a Zeiss Discovery V8 stereomicroscope (Oberkochen, Germany). Micro-morphological features were examined by mounting fungal structures in tap water. Observations and photographic documentation of conidiogenous cells and conidia were performed using an Olympus BX51 light microscope (Tokyo, Japan) equipped with differential interference contrast (DIC). Measurements for all taxonomically informative structures were reported as (minimum–) (mean – standard deviation) – (mean + standard deviation) (–maximum), with the mean and standard deviation calculated from at least 50 individual measurements.

### DNA extraction, PCR amplification, and sequencing

Genomic DNA was extracted from fresh fungal mycelium scraped from the surface of 2-week-old cultures using the standard CTAB extraction protocol. To resolve the phylogenetic placement of the isolates within *Parafenestella*, five nuclear loci were amplified: the internal transcribed spacer (ITS) region, the large subunit (LSU) of the ribosomal DNA, the RNA polymerase II second largest subunit (*rpb2*), the translation elongation factor 1-alpha (*tef1*), and the beta-tubulin (*tub2*) gene. Polymerase chain reactions (PCR) were conducted using the following standard primer pairs: ITS5/ITS4 for the ITS region (White et al. 1990); LR0R/LR5 for LSU (Vilgalys and Hester 1990); fRPB2-5F/fRPB2-7cR for *rpb2* (Liu et al. 1999); 2218F/983R for *tef1* (Rehner and Buckley 2005); and T1/Bt2b for *tub2* (O’Donnell and Cigelnik 1997). The PCR thermal cycling conditions followed those described by Su et al. (2022). Amplicons were visualized on a 1% agarose gel, subsequently purified, and sequenced by a commercial sequencing facility.

### Phylogenetic analyses

Newly generated sequences were assembled, trimmed, and subjected to BLASTn searches against the NCBI GenBank database to identify the closest matching taxa. Based on recent taxonomic reassessments of the fenestelloid clades, reference sequences for *Parafenestella* species and selected outgroups were downloaded and included in the dataset. Alignments for each genetic locus were generated using MAFFT and manually adjusted in MEGA v.7 (Kumar et al. 2016) as needed. The five individual datasets were then concatenated into a multi-locus supermatrix using SequenceMatrix v.1.7.8.

Maximum Likelihood (ML) and Bayesian Inference (BI) analyses were performed to infer evolutionary relationships. The ML analysis was conducted using IQ-TREE (Nguyen et al. 2015). The best-fit nucleotide substitution models for each gene partition were selected using ModelFinder (Kalyaanamoorthy et al. 2017) implemented within IQ-TREE, based on the Bayesian Information Criterion (BIC). Nodal support was assessed using 1,000 ultrafast bootstrap replicates (Hoang et al. 2018). Bayesian Inference (BI) was performed using MrBayes v.3.2.6 (Ronquist et al. 2012). Two independent Markov Chain Monte Carlo (MCMC) runs were executed for 5,000,000 generations, with trees sampled every 1,000^th^ generation. The initial 25% of sampled trees were discarded as burn-in, and the remaining trees were used to calculate the majority-rule consensus tree and posterior probabilities (PP). The resulting phylograms were visualized in FigTree v.1.4.2 (Rambaut 2014) and finalized using Adobe Illustrator.

## Results

### Phylogenetic analyses

**Figure 1.**
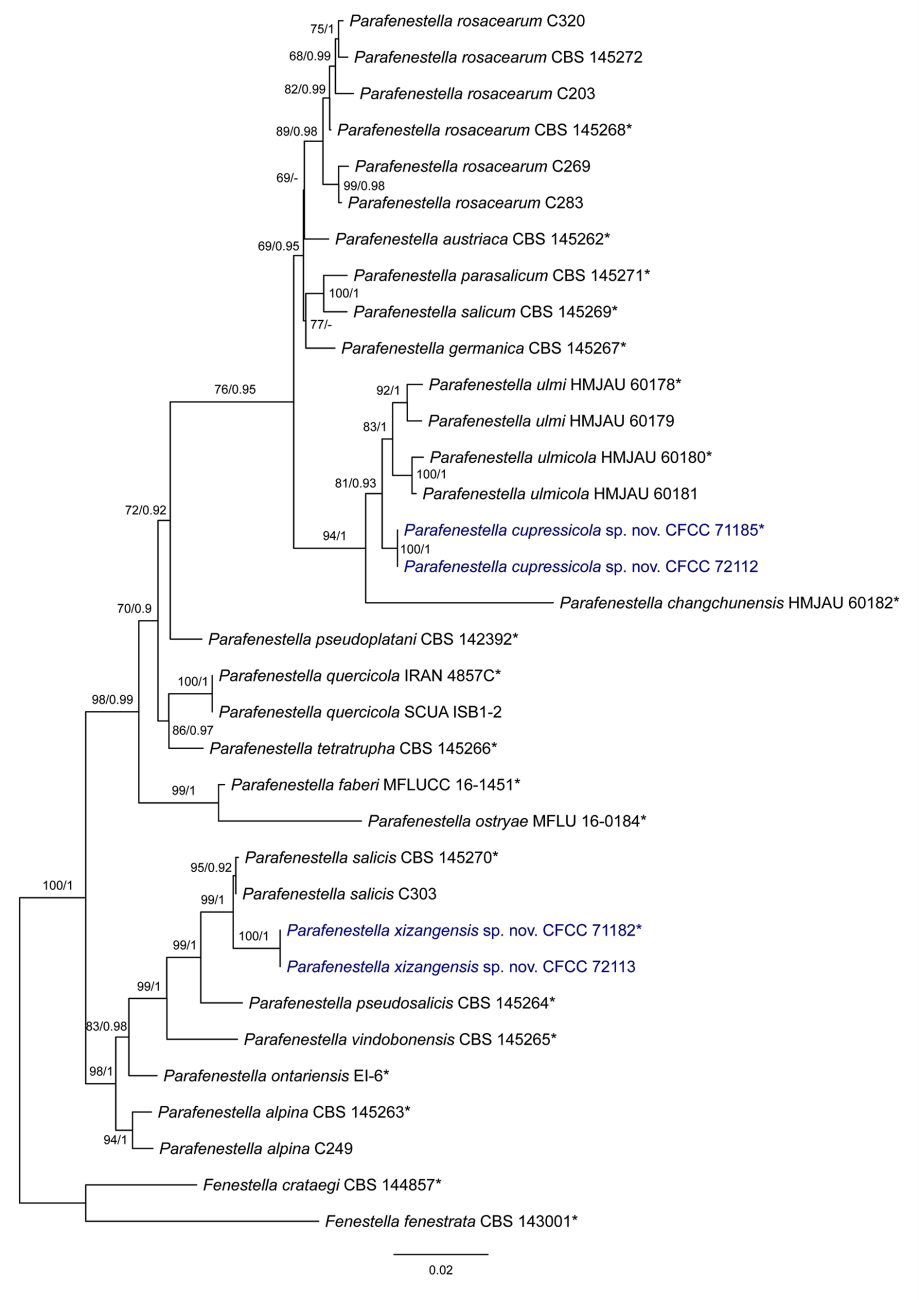
Maximum likelihood tree of *Parafenestella* generated from combined ITS, LSU, *rpb2*, *tef1*, and *tub2* sequence data. Bootstrap support values ≥ 50% and Bayesian posterior probabilities ≥ 0.90 are demonstrated at the branches. *Fenestella crataegi* (CBS 144857) and *F. fenestrata* (CBS 143001) represent the outgroup taxa. Isolates from the present study are indicated in blue and * means ex-type strains.

The concatenated five-gene dataset (ITS, LSU, *rpb2*, *tef1*, and *tub2*) used to resolve the phylogeny of *Parafenestella* included 34 strains (Table 1). *Fenestella crataegi* (CBS 144857) and *F. fenestrata* (CBS 143001) were utilized as the outgroup taxa. The final alignment consisted of 4177 characters (including gaps), with the following partition lengths: ITS (1–575 bp), LSU (576–1425 bp), *rpb2* (1426–2281 bp), *tef1* (2282–3503 bp), and *tub2* (3504–4177 bp). The best-scoring ML tree (Fig. 1) yielded a final likelihood value of −18341.77. According to the Bayesian Information Criterion (BIC), ModelFinder selected the following best-fit models: TNe+G4 for ITS, K2P+I for LSU, K3P+G4 for *rpb2*, TN+F+R2 for *tef1*, and TN+F+I+G4 for *tub2*. Phylogenetic inferences using both ML and BI yielded nearly identical topologies.

**Table 1.** Taxa information and GenBank accession numbers used in the phylogenetic analyses.

**Species**	**Strain**	**GenBank accession no**.					**Reference**
		**ITS**	**LSU**	***rpb2***	***tef1***	***tub2***	
*Fenestella crataegi*	CBS 144857*	MK356282	MK356282	MK357512	MK357555	MK357599	Jaklitsch and Voglmayr 2020
*F. fenestrata*	CBS 143001*	MF795765	MF795765	MF795807	MF795853	MF795893	Jaklitsch et al. 2018
*Parafenestella alpina*	CBS 145263*	MK356302	MK356302	MK357530	MK357574	MK357615	Jaklitsch and Voglmayr 2020
*P. alpina*	C249	MK356303	MK356303	MK357531	MK357575	MK357616	Jaklitsch and Voglmayr 2020
*P. austriaca*	CBS 145262*	MK356304	MK356304	MK357532	MK357576	MK357617	Jaklitsch and Voglmayr 2020
*P. changchunensis*	HMJAU 60182*	OL996119	OL897170	NA	OL944600	OL898719	Su et al. 2022
*P. cupressicola* sp. nov.	CFCC 71185*	PZ362984	PZ362980	PZ362184	PZ362188	PZ362192	This study
*P. cupressicola* sp. nov.	CFCC 72112	PZ362985	PZ362981	PZ362185	PZ362189	PZ362193	This study
*P. faberi*	MFLUCC 16-1451*	KY563071	KY563074	NA	NA	NA	Wanasinghe et al. 2017
*P. germanica*	CBS 145267*	MK356305	MK356305	MK357533	MK357577	MK357618	Jaklitsch and Voglmayr 2020
*P. ontariensis*	EI-6*	OM286882	OM286884	NA	NA	NA	Ilyukhin et al. 2022
*P. ostryae*	MFLU 16-0184*	KY563072	KY563075	NA	NA	NA	Wanasinghe et al. 2017
*P. parasalicum*	CBS 145271*	MK356306	MK356306	MK357534	MK357578	MK357619	Jaklitsch and Voglmayr 2020
*P. pseudoplatani*	CBS 142392*	MF795788	MF795788	MF795830	MF795876	MF795914	Jaklitsch et al. 2018
*P. pseudosalicis*	CBS 145264*	MK356307	MK356307	MK357535	MK357579	MK357620	Jaklitsch and Voglmayr 2020
*P. quercicola*	IRAN 4857C*	OR440609	NA	NA	OR450812	OR450818	Eisvand et al. 2024
*P. quercicola*	SCUA-ISB1-2	OR440610	NA	NA	OR450813	OR450819	Eisvand et al. 2024
*P. rosacearum*	CBS 145268*	MK356311	MK356311	MK357539	MK357583	MK357624	Jaklitsch and Voglmayr 2020
*P. rosacearum*	C203	MK356308	MK356308	MK357536	MK357580	MK357621	Jaklitsch and Voglmayr 2020
*P. rosacearum*	C269	MK356309	MK356309	MK357537	MK357581	MK357622	Jaklitsch and Voglmayr 2020
*P. rosacearum*	C283	MK356310	MK356310	MK357538	MK357582	MK357623	Jaklitsch and Voglmayr 2020
*P. rosacearum*	C320	MK356315	MK356315	MK357543	MK357587	NA	Jaklitsch and Voglmayr 2020
*P. rosacearum*	CBS 145272	MK356314	MK356314	MK357542	MK357586	MK357627	Jaklitsch and Voglmayr 2020
*P. salicis*	CBS 145270*	MK356317	MK356317	MK357545	MK357589	MK357629	Jaklitsch and Voglmayr 2020
*P. salicis*	C303	MK356316	MK356316	MK357544	MK357588	MK357628	Jaklitsch and Voglmayr 2020
*P. salicum*	CBS 145269*	MK356318	MK356318	MK357546	MK357590	MK357630	Jaklitsch and Voglmayr 2020
*P. tetratrupha*	CBS 145266*	MK356319	MK356319	MK357547	MK357591	MK357631	Jaklitsch and Voglmayr 2020
*P. ulmi*	HMJAU 60178*	OL996115	OL897166	OL944501	OL944596	OL898723	Su et al. 2022
*P. ulmi*	HMJAU 60179	OL996116	OL897167	OL944502	OL944597	OL898717	Su et al. 2022
*P. ulmicola*	HMJAU 60180*	OL996117	OL897168	OL944503	OL944598	OL898724	Su et al. 2022
*P. ulmicola*	HMJAU 60181	OL996118	OL897169	OL944504	OL944599	OL898718	Su et al. 2022
*P. vindobonensis*	CBS 145265*	MK356320	MK356320	MK357548	MK357592	MK357632	Jaklitsch and Voglmayr 2020
*P. xizangensis* sp. nov.	CFCC 71182*	PZ362986	PZ362982	PZ362186	PZ362190	PZ362194	This study
*P. xizangensis* sp. nov.	CFCC 72113	PZ362987	PZ362983	PZ362187	PZ362191	PZ362195	This study

Note. “NA” indicates unavailable sequences, sequences produced in the current study are in bold and * means ex-type strains.

In the multi-locus phylogenetic tree (Fig. 1), the four newly sequenced strains obtained from Xizang were distributed into two distinct lineages within the genus. The two strains representing *P. cupressicola* sp. nov. (CFCC 71185 and CFCC 72112), clustered together to form a highly supported separate clade (ML/BI = 100/1). Phylogenetically, *P. cupressicola* forms a sister lineage to the clade comprising *P. ulmi* (HMJAU 60178, HMJAU 60179) and *P. ulmicola* (HMJAU 60180, HMJAU 60181). Similarly, the two strains representing *P. xizangensis* sp. nov. (CFCC 71182 and CFCC 72113), clustered into a distinct and well-supported monophyletic clade (ML/BI = 100/1). This species is shown to be closely related to *P. salicis* (CBS 145270, C303), forming a sister relationship with high statistical support (ML/BI = 99/1). The phylogenetic results strongly support the establishment of *P. cupressicola* and *P. xizangensis* as two independent and novel species within *Parafenestella*.

### Taxonomy

#### Parafenestella cupressicola

Taxon classificationFungiFenestridaFenestellidae

Y.M. Liang & Ning Jiang sp. nov.

DC4835D6-8B16-5666-BCA2-F52227BD4AD5

863593

Fig. 2

##### Etymology.

Named after the host genus *Cupressus* and “-*cola*” = “inhabiting”.

**Figure 2.**
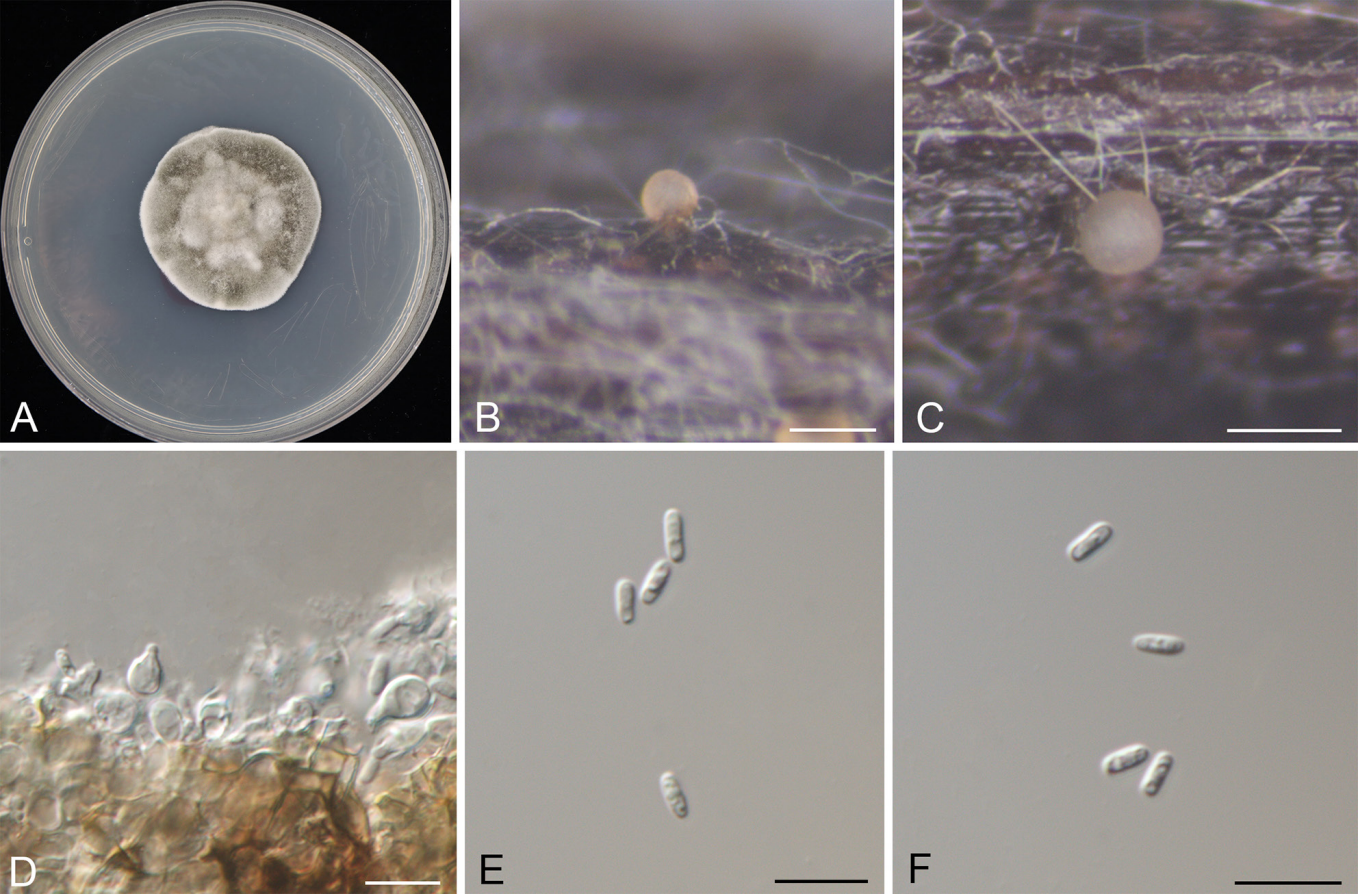
Morphology of *Parafenestella cupressicola*. **A**. Colony on PDA after 2 weeks; **B, C**. Conidiomata in pine needles on SNA; **D**. Conidiogenous cells; **E, F**. Conidia. Scale bars: 200 µm (**B, C**); 10 µm (**D–F**).

##### Description.

***Colonies*** on PDA flat, spreading, with abundant flocculent aerial mycelium and entire margin, first white, turning hazel, slow growing, reaching 39 mm diam after 2 weeks at 25 °C, sterile. ***Colonies*** on SNA inconspicuous, forming conidiomata in pine needles after 6 weeks. ***Conidiomata*** pycnidial, scattered, immersed in substrate, conical to globose, 90–120 μm diam., 70–100 μm high, brown, with buff conidial drops. ***Peridium*** thin, pseudoparenchymatous, olivaceous. ***Conidiophores*** reduced to conidiogenous cells. ***Conidiogenous cells*** phialidic, subglobose to globose, 3.5–5 × 3–4.5 μm. ***Conidia*** hyaline, oblong to ellipsoid, thin-walled, aseptate, (3–)3.5–4(–4.5) × 1.5–2 (av. = 3.8 ± 0.4 × 1.6 ± 0.2, n = 50) μm, L/W ratio = 2.2–2.6.

##### Materials examined.

China • Xizang Autonomous Region (Tibet), Linzhi City, Lang County, 29°4'4"N, 93°4'36"E, 3093 m asl, from a healthy cone of *Cupressus gigantea*, 5 Jun. 2024, *Ning Jiang, Jiangrong Li, Jieting Li & Liangna Guo* (holotype CAF800152, dried culture, ex-holotype cultures CFCC 71185, CFCC 72112).

##### Notes.

*Parafenestella cupressicola* sp. nov. is phylogenetically closely related to *P. ulmi* and *P. ulmicola* (Fig. 1). However, *P. cupressicola* differs from *P. ulmi* by significantly shorter conidiogenous cells (3.5–5 μm in *P. cupressicola* vs. 18–24 μm in *P. ulmi*); and from *P. ulmicola* by larger conidia (3.5–4 × 1.5–2 μm in *P. cupressicola* vs. 1.4–2.5 × 0.6–0.9 μm in *P. ulmicola*) (Su et al. 2022). In addition, *P. cupressicola* is molecularly distinguished from *P. ulmi* (3/510 bp in ITS, 1/851 bp in LSU, 8/856 bp in *rpb2*, 4/315 bp in *tub2*) and *P. ulmicola* (3/510 bp in ITS, 1/851 bp in LSU, 9/676 bp in *rpb2*, 6/315 bp in *tub2*) (Su et al. 2022).

#### Parafenestella xizangensis

Taxon classificationFungiFenestridaFenestellidae

Y.M. Liang & Ning Jiang sp. nov.

29AB630A-B9EE-5A38-9CC3-CEC3382502F6

863594

Fig. 3

##### Etymology.

Named after the collection site, the Xizang Autonomous Region.

**Figure 3.**
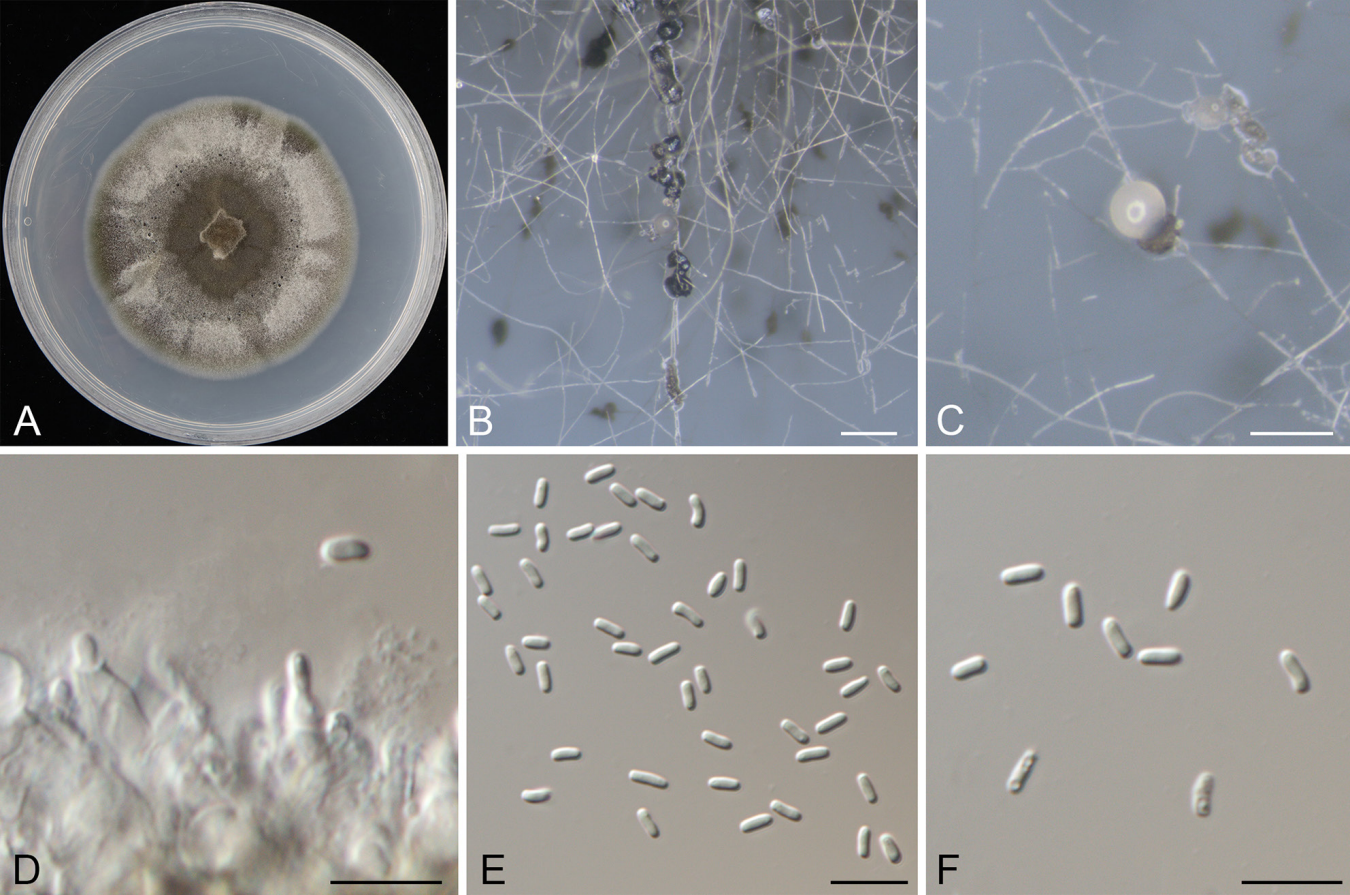
Morphology of *P. xizangensis*. **A**. Colony on PDA after 2 weeks; **B, C**. Conidiomata on SNA; **D**. Conidiogenous cells; **E, F**. Conidia. Scale bars: 200 µm (**B, C**); 10 µm (**D–F**).

##### Description.

***Colonies*** on PDA flat, spreading, with thin flocculent aerial mycelium and entire margin, first gray, turning dark brick, slow growing, reaching 62 mm diam after 2 weeks at 25 °C, sterile. ***Colonies*** on SNA inconspicuous, forming conidiomata after 4 weeks. ***Conidiomata*** pycnidial, gregarious, globose, 65–110 μm diam., 55–95 μm high, dark brown, with whitish conidial drops. ***Peridium*** thin, pseudoparenchymatous, olivaceous. ***Conidiophores*** reduced to conidiogenous cells. ***Conidiogenous cells*** phialidic, lageniform, 6.5–13 × 3.5–6 μm. ***Conidia*** hyaline, oblong to ellipsoid, thin-walled, aseptate, 3.5–4(–4.5) × (1–)1.5(–2) (av. = 3.8 ± 0.2 × 1.5 ± 0.2, n = 50) μm, L/W ratio = 2.1–3.1.

##### Materials examined.

China • Xizang Autonomous Region (Tibet), Linzhi City, Lang County, 29°3'9"N, 92°57'22"E, 3163 m asl, from a healthy branch of *Rosa sericea*, 5 Jun. 2024, *Ning Jiang, Jiangrong Li, Jieting Li & Liangna Guo* (holotype CAF800153, dried culture, ex-holotype cultures CFCC 71182, CFCC 72113).

##### Notes.

*Parafenestella xizangensis* sp. nov. is phylogenetically closely related to *P. salicis* (Fig. 1). These two species are similar in conidial size (3.5–4 × 1.5 μm in *P. xizangensis* vs. 3.7–4.5 × 1.1–1.5 μm in *P. salicis*) (Jaklitsch and Voglmayr 2020). However, they can be distinguished by nucleotide differences: ITS (31/529 bp), *tef1* (2/274 bp), and *tub2* (7/335 bp) (Jaklitsch and Voglmayr 2020).

## Discussion

In this study, we describe and document two novel endophytic species of *Parafenestella*, *P. cupressicola* and *P. xizangensis*, discovered in the high-altitude forests of Xizang, China. Since Jaklitsch et al. (2018) redefined the family *Cucurbitariaceae* and established the genus *Parafenestella*, the known taxonomic diversity and ecological plasticity of this genus have expanded rapidly. Initially, *Parafenestella* was considered primarily fungicolous, obligately colonizing the fruiting bodies of *Diaporthales* in temperate regions (Jaklitsch et al. 2018; Jaklitsch and Voglmayr 2020). Subsequent surveys progressively revealed saprobic species, such as *P. changchunensis*, *P. ulmi*, and *P. ulmicola* from woody litter in northeast China (Su et al. 2022). Our findings further expand the ecological repertoire of *Parafenestella* by providing the first evidence of its endophytic capability in woody plants.

Morphologically, species delimitation within *Cucurbitariaceae* using asexual morphs is notoriously difficult. Previous studies have highlighted that the *phoma*- or *pyrenochaeta*-like asexual morphs of fenestelloid fungi are highly convergent and generally lack sufficient traits for reliable species-level distinction (Aveskamp et al. 2010; Jaklitsch and Voglmayr 2020). However, our precise morphometric evaluations demonstrate that distinct micro-morphological differences can still be resolved. For instance, despite their close phylogenetic affinity, *P. cupressicola* exhibits significantly shorter conidiogenous cells than its sister taxa, *P. ulmi*, and distinctly larger conidia than *P. ulmicola* (Su et al. 2022).

Given the limitations of traditional morphology, molecular phylogeny is indispensable. Jaklitsch and Voglmayr (2020) emphasized that the complex phylogenetic structure of the fenestelloid clades can only be robustly resolved using protein-coding genes. Consistent with this, recent Assemble Species by Automatic Partitioning (ASAP) analyses identified *tub2* as the optimal DNA barcode for *Parafenestella*, effectively delimiting closely related cryptic species where ribosomal markers (ITS and LSU) fail (Su et al. 2022). Our concatenated five-gene (ITS, LSU, *tub2*, *tef1*, *rpb2*) phylogenetic analyses provided high statistical support for the independence of *P. cupressicola* and *P. xizangensis*. Furthermore, the significant infraspecific genetic variation observed within *Parafenestella* suggests that the genus is undergoing active speciation (Jaklitsch and Voglmayr 2020).

The discovery of these endophytic species aligns with the broader recognition of Xizang as a critical biodiversity hotspot for forest microfungi. Systematic investigations led by our research group have recently revealed an extraordinary diversity of fungal taxa associated with woody plants in this region. Significant taxonomic advancements have been made in documenting forest pathogens, including numerous novel species of *Cytospora* associated with branch cankers (Jiang et al. 2025; Li et al. 2024, 2025), *Diaporthe* associated with *Alnus nepalensis* leaf spots and cankers (Li et al. 2025), and *Sporocadus* from various forest ecosystems (Li et al. 2026). While these previous studies primarily focused on pathogenic fungi from symptomatic tissues, the discovery of *Parafenestella* from healthy tissues underscores the functional complexity. This parallel existence of diverse pathogens and endophytes suggests that the Tibetan Plateau hosts a highly specialized mycobiota that has evolved complex ecological strategies, ranging from aggressive pathogenicity to latent endophytism, to adapt to extreme alpine environments.

In conclusion, the discovery of *P. cupressicola* and *P. xizangensis* not only enriches the known biodiversity of *Cucurbitariaceae* but also fills a critical geographical gap in high-altitude habitats. Future investigations incorporating comparative genomics and extensive sampling of alpine endophytes will be crucial to fully unravel the mechanisms underlying the geographic divergence, host co-evolution, and ecological adaptability of the fenestelloid fungi.

## Supplementary Material

XML Treatment for Parafenestella cupressicola

XML Treatment for Parafenestella xizangensis

## Additional information

### Artificial Intelligence (AI) use

The authors accept full responsibility for the content of the manuscript, including the disclosure of any use of AI.

No AI tools were used in the preparation of this manuscript.

### Author ORCIDs

K. Pang https://orcid.org/0009-0006-1132-0802

Y. Wu https://orcid.org/0009-0007-5095-2738

Y. Liang https://orcid.org/0000-0002-1690-5512

N. Jiang https://orcid.org/0000-0002-9656-8500

### Data availability

All of the data that support the findings of this study are available in the main text.
